# Avidity engineering of human heavy-chain-only antibodies mitigates neutralization resistance of SARS-CoV-2 variants

**DOI:** 10.3389/fimmu.2023.1111385

**Published:** 2023-02-21

**Authors:** Wenjuan Du, Rick Janssens, Anna Z. Mykytyn, Wentao Li, Dubravka Drabek, Rien van Haperen, Marianthi Chatziandreou, Melanie Rissmann, Joline van der Lee, Melissa van Dortmondt, Itziar Serna Martin, Frank J. M. van Kuppeveld, Daniel L. Hurdiss, Bart L. Haagmans, Frank Grosveld, Berend-Jan Bosch

**Affiliations:** ^1^ Virology Section, Infectious Diseases and Immunology Division, Department of Biomolecular Health Sciences, Faculty of Veterinary Medicine, Utrecht University, Utrecht, Netherlands; ^2^ Department of Cell Biology, Erasmus Medical Center, Rotterdam, Netherlands; ^3^ Harbour BioMed, Rotterdam, Netherlands; ^4^ Department of Viroscience, Erasmus Medical Center, Rotterdam, Netherlands

**Keywords:** heavy-chain-only antibody, avidity, SARS-CoV-2, antibody-mediated neutralization, neutralization escape

## Abstract

Emerging SARS-CoV-2 variants have accrued mutations within the spike protein rendering most therapeutic monoclonal antibodies against COVID-19 ineffective. Hence there is an unmet need for broad-spectrum mAb treatments for COVID-19 that are more resistant to antigenically drifted SARS-CoV-2 variants. Here we describe the design of a biparatopic heavy-chain-only antibody consisting of six antigen binding sites recognizing two distinct epitopes in the spike protein NTD and RBD. The hexavalent antibody showed potent neutralizing activity against SARS-CoV-2 and variants of concern, including the Omicron sub-lineages BA.1, BA.2, BA.4 and BA.5, whereas the parental components had lost Omicron neutralization potency. We demonstrate that the tethered design mitigates the substantial decrease in spike trimer affinity seen for escape mutations for the hexamer components. The hexavalent antibody protected against SARS-CoV-2 infection in a hamster model. This work provides a framework for designing therapeutic antibodies to overcome antibody neutralization escape of emerging SARS-CoV-2 variants.

## Introduction

Antibodies are crucial components of the humoral immune system against SARS-CoV-2 infection and can be developed into powerful therapeutics to fight COVID-19 (1). Neutralizing antibodies target the SARS-CoV-2 spike (S) protein, a class I fusion protein which mediates virus-cell entry. The S protein forms a homotrimer and is divided into a membrane-distal S1 subunit and a membrane-anchored S2 subunit that mediates fusion of the viral and cellular membranes. The S1 subunit can be further divided into an N-terminal domain (NTD) that may engage attachment factors (2–5) and the receptor binding domain (RBD) that binds the human ACE2 receptor (6, 7). The RBD in the S protein homotrimer can adopt an open (up) or closed (down) conformation, with only the open RBD able to engage the ACE2 receptor. The NTD and RBD are the major targets of potent neutralizing antibodies (8–11). Four major antibody classes in the RBD have been structurally defined, in which class 1 and 2 epitopes overlap with the ACE2-binding site while class 3 and 4 epitopes are outside the ACE2-binding site (11). Contrary to the RBD, most neutralizing antibodies that recognize the NTD target a single antigenic supersite composed of multiple loops (8).

SARS-CoV-2 variants of concern (VOCs) such as Beta, Gamma and in particular Omicron and its sublineages carry S mutations that reduce or abolish neutralization potency of many antibodies, including all antibodies that were emergency authorized for therapeutic use (12–17). These mutations concentrate in the epitopes in the S protein NTD and RBD targeted by neutralizing antibodies lowering their binding affinity and neutralization potency. Thus, strategies to develop antibodies that can resist viral escape are needed. Rationally designed antibody cocktails that cover non-overlapping epitopes might expand coverage of SARS-CoV-2 variants (18, 19), however such an approach increases manufacturing costs and demands higher dosing.

Alternative approaches – including the generation of multispecific antibodies – have been pursued to generate anti-SARS-CoV-2 spike antibodies with increased neutralization breadth (20–24). The binding capacity of antibodies to two or more unique spike epitopes mitigates the risk of neutralization escape by variants. Conventional antibodies require the expression of a heavy and light chain which complicates the development of multispecific antibodies. The single-chain format of single-domain antibodies (sdAbs) greatly facilitates engineering of multimeric and multispecific antibodies with increased valency (25–33). SdAbs are 15 kDa in size and derived from the variable domain (VH) of heavy-chain-only antibodies (HCAbs). These HCAbs are devoid of light chains and lack the CH1 domain in the heavy chain and are naturally found in camelids and sharks. Increasing valency of sdAbs (21, 26, 34–36) can enhance the apparent affinity (known as avidity) for target antigens and several formats have been used to increase valency of single domain antigen binding domains including domain linking (22–24, 29–32, 37), fusion with human dimeric Fc fragments (21, 26, 32) or alternative self-assembling multimerization tags (28, 38). These strategies have been successfully employed to increase neutralization potency and/or breadth of sdAbs against influenza virus (39, 40) and respiratory syncytial virus (41). Avidity engineering of SARS-CoV-2 sdAbs resulted in antibodies with exceptional avidity and ultrahigh neutralization potency (21, 24, 28–30, 32, 37, 38, 42–44), yet the promise of this approach to counteract neutralization escape of antigenically drifted SARS-CoV-2 variants has been poorly explored (21).

To generate SARS-CoV-2 antibodies with increased neutralization potency and breadth, we generated mono- or multispecific heavy-chain-only antibodies of human origin carrying either one, two or three sdAbs. We utilized the antibody repertoire of SARS-CoV-2 S immunized, transgenic mice expressing human HCAbs (45, 46) that consist of a human VH domain directly linked to the human IgG1 constant domains CH2/CH3 that form the dimeric Fc region. Based on a collection of SARS-CoV-2 neutralizing HCAbs targeting distinct S protein epitopes we generated a collection of tetravalent and hexavalent antibodies by linking additional VH domains to the N- and/or C-termini of the parental bivalent HCAb. A hexavalent biparatopic heavy-chain-only antibody integrating three antigen binding domains exhibited remarkable broad neutralization capacity against SARS-CoV-2 VOCs, including Omicron BA.1, BA.2, BA.2.12.1 and BA.4/BA.5, whereas the parental HCAbs had lost neutralization potency against these variants. Prophylactic administration of this antibody confers protection of hamsters against SARS-CoV-2 challenge. Overall, our findings indicate that antibody engineering to increase valency and binding modalities can be a promising approach to overcome neutralization resistance by SARS-CoV-2 variants.

## Methods

### Viruses and cells

Calu-3 cells were cultured in Opti-MEM I (1X) + GlutaMAX (Gibco) supplemented with 10% fetal bovine serum (FBS, Biowest, France), penicillin (100 IU/ml), and streptomycin (100 IU/ml). Human embryonic kidney (HEK) 293T and VeroE6 cells were maintained in Dulbecco’s modified Eagle’s medium (DMEM) supplemented with 10% FBS (Biowest), sodium pyruvate (1 mM; Gibco, CA, USA), nonessential amino acids (1×; Lonza, Bornem, Belgium), penicillin (100 IU/ml), and streptomycin (100 IU/ml). Cell lines were kept at 37°C in a humidified CO_2_ incubator. Cell lines were tested negative for mycoplasma. Calu-3 cells were used to grow SARS-CoV-2 isolates for three passages. Infections were carried out at a multiplicity of infection of 0.01 for stock production, and virus was collected at 72 hours after infection, clarified by centrifugation, and stored at −80°C in aliquots until use. All experiments with infectious SARS-CoV-2 was performed in a Class II Biosafety Cabinet under BSL-3 conditions at Erasmus Medical Center. Viral genome sequences were determined using Illumina deep sequencing as described before (47). The 614G virus (clade B; isolate Bavpat-1; European Virus Archive Global #026 V-03883) passage 3 sequence was identical to the passage 1 (provided by Dr. Christian Drosten). The Alpha (B.1.1.7; MW947280), Gamma (P.1; OM442897), Delta (B.1.617.2; OM287123), Omicron BA.1(B.1.1.529.1; OM287553), Omicron BA.2 (B.1.1.529.2), Omicron BA.4 (B.1.1.529.4), Omicron BA.5 (B.1.1.529.5) variant passage 3 sequences were identical to the original respiratory specimens. Low coverage regions in the spike gene were confirmed by Sanger sequencing. The Beta variant (B.1.351; OM286905) passage 3 sequence contained two mutations compared with the original respiratory specimen: one synonymous mutation C13860T (Wuhan-Hu-1 position) in ORF1ab and a L71P change in the E gene (T26456C, Wuhan-Hu-1 position). S protein mutations found in SARS-CoV-2 variants used, relative to ancestral SARS-CoV-2 are listed in **Figure S3**.

### Expression and purification of SARS-CoV-2 S proteins

Human codon-optimized genes were synthesized at GenScript encoding the 6P- or 2P-stabilized SARS-CoV-2 S ectodomain expression construct (S protein residues 1 to 1213, Wuhan-Hu-1 strain) with a C-terminal T4 foldon trimerization motif, followed by a Twin-Strep-tag (48). Constructs encoding the S1 (residues 1 to 682), NTD (residues 1 to 294), or RBD (residues 329 to 538) of SARS-CoV-2 S (Wuhan-Hu-1) were C-terminally tagged with Strep-tag affinity tag (48). Expression construct with the human codon-optimized gene encoding the S1 protein (residues 1 to 679) of Omicron BA.1 (B.1.1.529.1) was generated including a C-terminal Strep-tag. All proteins were expressed transiently in HEK-293T [American Type Culture Collection (ATCC), CRL-11268] cells from pCAGGS expression plasmids, and secreted proteins were purified from culture supernatants using streptactin beads (IBA, Göttingen, Germany) following the manufacturer’s protocol. S variants with single-site residue substitutions or deletions were generated by PCR based site-directed mutagenesis.

### Generation of HCAbs against SARS-CoV-2 S

Ten Harbour HCAb transgenic mice (v2.1 9VH3) were immunized with the 2P-stabilized trimeric SARS-CoV-2 (Wuhan-Hu-1) spike protein according to the approved animal license AVD101002016512 and the study plan 16-512-20 in Erasmus MC animal facility. Antigen-specific blood titers were followed during the immunization process by antigen-specific ELISA. Seven mice showing satisfactory titers (saturation signal for plasma dilution 1:3000 and higher) were used for making HCAbs libraries. Selected animals were sacrificed, and their lymphoid organs (lymph nodes, spleen and bone marrow) were collected. Antigen-specific B cells and plasma cells were used for the purification of total RNA, followed by reverse transcription and cDNA synthesis and the amplification of human VH regions. Antigen-specific B cells were isolated by magnetic separation of B cells bound to the biotinylated trimeric spike protein on Dynabeads M-280 streptavidin, while CD138 positive plasma cells were isolated using Miltenyi Biotec CD138 plasma cell isolation kit according to the manufacturer’s instruction. All procedures were described in detail previously (45). In short, VHs were cloned as PvuII/BstEII fragments into PvuII/BstEII of the pCAG hygro hG1 vector (Harbour Ab). This was followed by the transformation of *E. coli* electro-competent cells (MegaX DH10B T1, Invitrogen). Single colonies were picked and grown in 96-well plates. Individual DNA plasmids were extracted and used to transfect HEK-293T cells in the same format transiently. Supernatants are tested for binding using ELISA. The DNA corresponding to selected positive clones is sequenced, and medium-scale production of original fully human bi-valent HCAbs is performed by transient transfection of HEK-293T cells with the same DNA plasmid. HCAbs are purified using Protein A affinity columns. For tetravalent HCAbs, VH3 was fused to VH1 *via* (GGGGS)_5_ linker, while the VH2 was linked to CH3 domain *via* an artificial hinge (ASERKPPVEPPPPP). The same linker and artificial hinge were applied to hexavalent HCAbs. Antibodies were expressed in HEK-293T cells after transient transfection using polyethylenimine with expression plasmids. Transfection mixture was replaced by 293 SFM II expression medium (Invitrogen, Carlsbad, CA, USA) 18 hours post transfection, supplemented with sodium bicarbonate (3.7 g/l), glucose (2.0 g/liter), Primatone RL-UF (3.0 g/liter), penicillin (100 IU/ml), streptomycin (100 IU/ml), GlutaMAX, and 1.5% dimethyl sulfoxide. Tissue culture supernatants were harvested 5 to 6 days after transfection, from which antibodies were purified using Protein A Sepharose (Thermo Fisher Scientific, Ireland) according to the manufacturer’s instructions.

### ELISA-based binding analysis of HCAbs and S antigens

Purified S antigens (1 µg/ml) or bi-, tetra-, and hexa-valent HCAbs (37.5 nM) were coated onto 96-well NUNC Maxisorp plates (Thermo Fisher Scientific) at 4°C overnight, followed by three washing steps with phosphate-buffered saline (PBS) containing 0.05% Tween 20. Plates were blocked with blocking buffer (PBS containing 3% bovine serum albumin [BSA; Fitzgerald, Acton, MA, USA] and 0.1% Tween 20) at room temperature (RT) for 2 hours. HCAbs or S antigens were allowed to bind to the plates at fivefold or fourfold serial dilutions, starting at 5 µg/ml (HCAbs) or 0.975 mM (S antigens) diluted in blocking buffer at RT for 1 hour. HCAb binding to the S proteins was determined using a 1:2000 diluted HRP-conjugated goat anti-human IgG (ITK Southern Biotech, Uden, NL) for 1 hour at RT. S antigen binding to HCAbs was determined using a 1:4000 diluted StrepMAB-Classic HRP (IBA). HRP activity was measured at 450 nm using tetramethylbenzidine substrate (BioFX, Eden Prairie, MN, USA) and an ELISA plate reader (EL-808, BioTek, Bornem, Belgium).

### ACE2 receptor binding inhibition assay

The ACE2 receptor binding inhibition assay was conducted as described previously (49). Briefly, purified soluble ACE2 (10 µg/ml) was coated onto 96-well NUNC Maxisorp plates (Thermo Fisher Scientific) at 4°C overnight, followed by three washing steps with PBS containing 0.05% Tween 20. Plates were blocked with 5% skim-milk (Nutricia, Amsterdam, NL) in PBS at RT for 2 hours. Fourfold serial dilutions of HCAbs starting at 200nM were pre-incubated with 200nM SARS-CoV-2 S RBD at RT for 2 h. HCAb-S mixtures were subsequently added to ACE2-coated plate and incubated at 4°C for 3 h. The binding of SARS-CoV-2 S RBD to ACE2 was detected using a 1:4000 diluted StrepMAB-Classic HRP (IBA) that recognizes the Strep-tag fused to SARS-CoV-2 S RBD domain. HRP activity was quantified using tetramethylbenzidine substrate (BioFX) and an ELISA plate reader at 450 nm. The percentage of SARS-CoV-2 S RBD binding to ACE2 was calculated as the ratio of the binding signal in the presence of HCAbs normalized to binding signal in the absence of HCAbs.

### BLI-based binding competition assay

Binding competition assay was carried out using biolayer interferometry (Octet Red348, Sartorius, USA), as described previously (49). In brief, SARS-CoV-2 S ectodomain trimer (50 µg/ml) was immobilized onto the Protein A biosensor (Sartorius, USA) *via* an anti-Streptag mAb (IBA). After a brief washing step in PBS, the biosensors were dipped into a well containing the primary HCAb (50 µg/ml) for 15 min, followed by a short washing step in PBS. Subsequently the biosensors were immersed into a well containing the HCAb 2 (50 µg/ml) for 15 min.

### Affinity determination *via* BLI

HCAb (50 µg/ml) was loaded to Protein A biosensor (Sartorius, USA) for 6 min. Antigen binding was monitored by incubating with twofold dilutions of recombinant SARS-CoV-2 S ectodomain trimer for 12 or 15 min, followed by a long dissociation step (30 min). All experiments were performed in Dulbecco’s PBS with Calcium and Magnesium (Lonza) at 30°C and with sensors shaking at 1000 rpm. The affinity constant K_D_ was calculated using a 1:1 Langmuir binding model using Fortebio Data Analysis 7.0 software.

### Pseudovirus neutralization assay

Human codon-optimized genes encoding the S proteins of SARS-CoV-2 of the ancestral Wuhan-Hu-1 virus (GenBank: NC_045512.2) or VOCs Alpha (B.1.1.7), Beta (B.1.351), Delta (B.1.617.2), Omicron BA.1(B.1.1.529.1), Omicron BA.2 (B.1.1.529.2), Omicron BA.2.12.1 (B.1.1.529.2.12.1) Omicron BA.4/5 (B.1.1.529.4/5) were synthesized by GenScript with a C-terminal 18-residue long cytoplasmic tail truncation (to increase cell surface expression levels), and cloned into the pCAGGS expression vector. Generation of SARS-CoV-2 S pseudotyped VSV and the neutralization assay was described previously (49). Briefly, HEK-293T cells at 70 to 80% confluency were transfected to express the SARS-CoV-2 S proteins. At 48 hours post transfection cells were infected with VSV G-pseudotyped VSVΔG harboring the firefly (Photinus pyralis) luciferase reporter gene. Twenty-four hours later, the supernatant was harvested, filtered through a 0.45µm filter, and stored at −80°C until use. SARS-CoV-2 S pseudotyped VSV was titrated on VeroE6 cells. In the virus neutralization assay, SARS-CoV-2 S pseudotyped virus (sufficient to generate 100,000 relative light units [RLU]) were mixed with an equal volume of threefold serially diluted HCAbs and incubated at RT for 1 hour. Virus-antibody mixtures were subsequently transferred to 96-well plate seeded VeroE6 cells, and further incubated at 37°C for twenty hours. VeroE6 cells were washed once with PBS and lysed with Passive lysis buffer (Promega). The expression of firefly luciferase was measured on a Berthold Centro LB 960 plate luminometer using d-luciferin as a substrate (Promega, Madison, WI, USA). The percentage of neutralization was calculated as the ratio of the reduction in RLU in the presence of HCAbs normalized to RLU in the absence of mAb. The IC50 values were determined using four-parameter logistic regression (GraphPad Prism v8.3.0).

### Authentic virus neutralization assay

HCAbs were tested for live virus neutralization using a plaque reduction neutralization (PRNT) assay. PRNT was performed according to a previously published protocol (47), with minor modifications. Briefly, 50 µl of serially diluted antibody in Opti-MEM I (IX) + GlutaMAX (Gibco, USA) was mixed 1:1 with virus (400 PFU) and incubated at 37°C for 1 hour. Subsequently the virus and antibody mixture was transferred to fully confluent monolayers of Calu-3 cells [washed once prior with Opti-MEM I (IX) + GlutaMAX]. After 8 hours of incubation, the cells were fixed with formalin, permeabilized with 70% ethanol, washed in PBS, and stained using rabbit anti–SARS-CoV nucleocapsid (1:2000 in 0.1% BSA in PBS; SinoBiological), followed by a goat anti-rabbit Alexa Fluor 488 antibody (1:2000 in 0.1% BSA in PBS; Invitrogen). Plates were scanned on the Amersham Typhoon Biomolecular Imager (GE Healthcare, USA). Data were analyzed using ImageQuantTL 8.2 image analysis software (GE Healthcare). The PRNT titer was calculated using GraphPad Prism 9, calculating a 50% reduction in infected cell counts based on nonlinear regression with bottom constraints of 0% and top constraints of 100%.

### Hamster challenge experiment

Female Syrian golden hamsters (Mesocricetus auratus; 6 weeks old; Janvier, France) were allowed to acclimatize to husbandry for at least 7 days. For unbiased experiments, all animals were randomly assigned to experimental groups. The first group of animals (*n*=8) was administered intraperitoneally with 10D12^VH1^-11C12^VH2^-10D12^VH3^ (10 mg/kg). Twenty-four hours after prophylactic treatment, those animals were inoculated intranasally with 1.0 x 10^4^ PFU of the Omicron BA.5 or Delta variants of SARS-CoV-2 (*n* = 4 each) in a total volume of 100 µl per animal. As negative control, a third group of animals was inoculated as mentioned before, but not treated (*n* = 4 Omicron BA.5; *n* = 4 Delta). On 4 dpi, all animals were euthanized, and the respiratory tract (lungs) was sampled for quantification of viral and genomic load.

Research involving animals was conducted in compliance with the Dutch legislation for the protection of animals used for scientific purposes (2014, implementing EU Directive 2010/63) and other relevant regulations. The licensed establishment where this research was conducted (Erasmus MC) has an approved OLAW Assurance # A5051-01. Research was conducted under a project license (2019–0075) from the Dutch competent authority and the study protocol (#17-4312) was approved by the institutional Animal Welfare Body. Animals were housed in groups of 2 animals in filter top cages (T3, Techniplast), in Class III isolators allowing social interactions, under controlled conditions of humidity, temperature and light (12-hour light/12-hour dark cycles). Food and water were available ad libitum. Animals were cared for and monitored (pre- and post-infection) daily by qualified personnel. The animals were anesthetized (3-5% isoflurane) for all invasive procedures. Hamsters were euthanized by cardiac puncture under isoflurane anesthesia and cervical dislocation.

### Cryo-EM grid preparation and data collection

To obtain a spike-HCAb complex for cryo-EM analysis, 80 μl of 4.2 mg/ml 6P stabilized S-ECD was combined with 20 μl of 10 mg/ml 10D12. The complex was incubated on ice for 5 minutes before being purified by size exclusion chromatography using a Superose^®^ 6 Increase 10/300 GL column, in buffer containing 20 mM Tris pH 8, 150 mM NaCl. The complex-containing fractions, as determined by SDS-PAGE, were pooled and concentrated to ~1 mg/ml. Approximately 3 µl of the sample was pipetted onto glow-discharged R1.2/1.3 200 mesh holey Cu carbon grids (Quantifoil) and then plunge-frozen in liquid ethane with a Vitrobot Mark IV (Thermo Fisher Scientific). Data were collected at the Netherlands Center for Electron Nanoscopy (NeCEN). Grids were loaded into a Titan Krios electron microscope (Thermo Fisher Scientific) operated at 300 kV, equipped with a K3 direct electron detector and Bioquantum energy filter (Gatan). The slit width of the energy filter was set to 20 eV. A total of 5018 movies were recorded in counting mode with EPU software (Thermo Fisher Scientific). Detailed data acquisition parameters are summarized in **Supplementary Table 1**.

### Cryo-EM image processing

Patch motion correction and patch CTF estimation were performed in cryoSPARC live (50). Micrographs with a CTF estimated resolution of worse than 10 Å were discarded, leaving 4997 images for further processing. The blob picker tool was then used to select 1215409 particles which were then extracted in a 100-pixel box (Fourier binned 4 × 4) and then exported to cryoSPARC for further processing. A single round of 2D classification was performed, after which 172971 particles were retained. Ab initio reconstruction generated two distinct conformations of the 10D12-bound spike, with either two or three RBDs in the open conformation. Particles corresponding to these two well defined classes were re-extracted in a 300-pixel box. During extraction, particles were Fourier binned by a non-integer value, resulting in a final pixel size of 1.1147 Å. Both particle stacks were then subjected to non-uniform refinement with either C1 or C3 symmetry imposed (51, 52), yielding S-HCAb reconstructions with global resolutions of 3.3 and 3.1 Å, respectively. After global refinement, a soft mask encompassing one RBD with the 10D12_VH_ bound was made in UCSF Chimera. Subsequently, each particle from the C3 symmetry–imposed reconstruction was assigned three orientations corresponding to its symmetry-related views using the symmetry expansion job. The soft mask was placed over a single RBD-10D12 region of the map, and the symmetry-expanded particles were subjected to masked 3D variability analysis (53). Local refinement was then performed on the particles belonging to the best resolved cluster, yielding a map with a global resolution of 4.1 Å. The “Gold Standard” Fourier shell correlation (FSC) criterion (FSC = 0.143) was used for resolution estimates. An overview of the data processing pipeline is shown in **Supplementary Figure S5**.

### Model building and refinement

To model the HCAb-SARS-CoV-2 spike glycoprotein interaction, the RBD crystal structure (residues 333-526; PDB ID 6M0J) (7) and an AlphFold2 (54) model of 10D12 were individually rigid-body fitted into the locally refined density map using the UCSF Chimera “Fit in map” tool (55). The two models were then combined and then subjected to automatic molecular dynamics flexible fitting using the Namdinator pipeline (56). Subsequently, an additional round of real space refinement in Phenix was performed (57), and the final model was validated with MolProbity (58).

### Analysis and visualization

Spike residues interacting with 10D12 were identified using PDBePISA (59). Figures were generated using UCSF ChimeraX (60). Structural biology applications used in this project were compiled and configured by SBGrid (61).

### Statistical analysis

Two-tailed nonparametric Mann-Whitney U tests and one-way analysis of variance (ANOVA) with Tukey’s multiple comparisons test were performed to analyze the statistical differences between two independent groups. A *p-*value of less than 0.05 was considered significant. Statistical analysis was done using GraphPad Prism 9.3.1.

## Results

**Figure 1:**
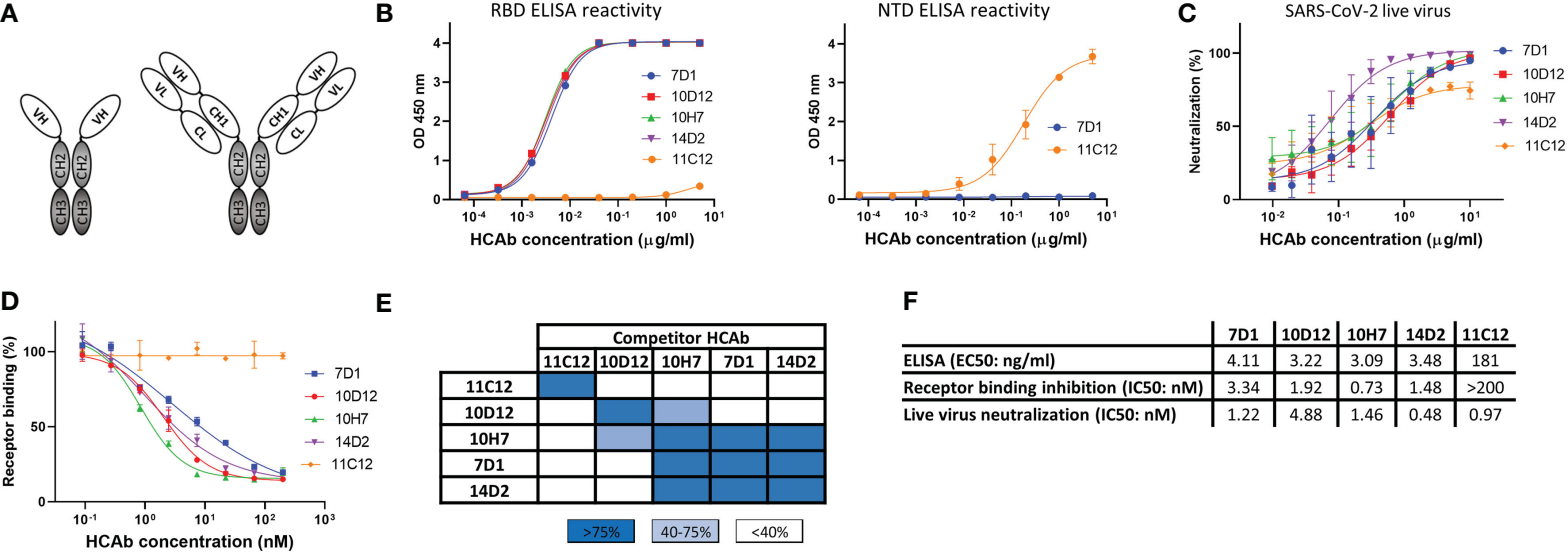
Human heavy-chain-only antibodies with SARS-CoV-2 neutralizing activity. **(A)** Schematic representation of a human heavy-chain-only antibody (HCAb) and a conventional IgG antibody; VH, variable heavy chain domain; VL, variable light chain domain; CL, constant light chain domain; CH1-CH3, constant heavy chain domains. CH2 and CH3 domains that comprise the Fc region are depicted in grey. **(B)** ELISA-reactivity of HCAbs to the receptor binding domain (RBD, left panel) and N-terminal domain (NTD, right panel) of the SARS-CoV-2 S protein. **(C)** Neutralization of SARS-CoV-2 by HCAbs in Calu-3 cells. **(D)** ACE2-receptor binding of the SARS-CoV-2 S ectodomain trimer was preincubated with each of the serially diluted HCAbs. Error bars indicate SD between at least two independent replicates. **(E)** Heatmap showing binding competition of HCAbs to the SARS-CoV-2 S ectodomain, as determined by biolayer interferometry. Results are classified using color shading codes with a percentage of inhibition ≥75% in blue, <75% but ≥40% in light blue, and no shading for a percentage of inhibition <40%. BLI sensorgrams showing the HCAb binding competition profiles are shown in **Supplementary Figure 1A**. Binding competition experiment was performed twice independently, data from one representative experiment are shown. **(F)** Antibody titers for ELISA-based binding, receptor binding inhibition and neutralizing potency of SARS-CoV-2 S-directed HCAbs were calculated based on inhibition curves shown in **(B, D, C)** respectively.

To construct a panel of candidate human VH domains for multimerization, HCAb mice (45, 46) were immunized with the S protein of SARS-CoV-2. Human heavy chain variable regions were PCR-amplified from the cDNAs generated from S protein-specific isolated B-cells from lymph nodes and plasma cells isolated from spleens and bone marrow (45). Two separate bacterial libraries were made by cloning VHs in a eukaryotic expression vector containing the human IgG1 backbone without the CH1 exon (**Figure 1A**). One thousand bacterial colonies from each library were cultured, and two thousand individual plasmids were purified and transfected into HEK-293T cells. Supernatants were screened in a SARS-CoV-2 S specific ELISA, and those positive in ELISA were further tested in a pseudovirus neutralization assay. Positive clones were sequenced, and unique fully human HCAbs were purified from transiently transfected HEK-293T cells.

From a panel of ~600 HCAbs with SARS-CoV-2 S ELISA-reactivity, of which ~150 displayed neutralizing activity against SARS-CoV-2 pseudovirus, we selected five HCAbs with a diverse range of epitopes in the S protein as candidates for multimerization. Four of these - 7D1, 10D12, 14D2 and 10H7 - bound the S protein RBD whereas 11C12 targeted the NTD (**Figure 1B**). All five HCAbs could neutralize infection of authentic SARS-CoV-2 into Calu-3 cells with IC50 values ranging from 0.48 to 4.88 nM (**Figures 1C, F**). In contrast to the NTD HCAb 11C12, all four RBD HCAbs prevented RBD from binding to ACE2 in a solid-phase assay, indicating that these HCAbs prevent infection by blocking viral attachment to target cells (**Figures 1D, F**). Sequence analysis of the VH regions of the five HCAbs indicated that 7D1, 10D12, 14D2 and 10H7 shared the heavy chain germline (IGHV3-53) origin but differed considerably in the somatic hypermutations and their complementarity determining region (CDR) 3 sequences, whereas 11C12 was derived from the IGHV3-48 germline (**Figure S1A**). We subjected the five HCAbs to mutual binding competition to SARS-CoV-2 S by biolayer interferometry (BLI) to study their target site. The HCAbs were found to target three distinct regions in S. 11C12 binds a distinct epitope in the S NTD. 7D1, 14D2 and 10H7 competed for the same binding site on RBD, whereas 10D12 binds to a different RBD region and could bind to the RBD at the same time as 7D1 and 14D2, with only partial interference in RBD binding seen for 10H7 (**Figures 1E**, **S1B**). BLI was used to assess the binding affinity of each HCAb to trimeric S with apparent binding affinities in the nanomolar range (25.5 to 213 nM) (**Figure S1C**).

### Increased valency of HCAbs potentiates neutralizing activity against SARS-CoV-2

**Figure 2:**
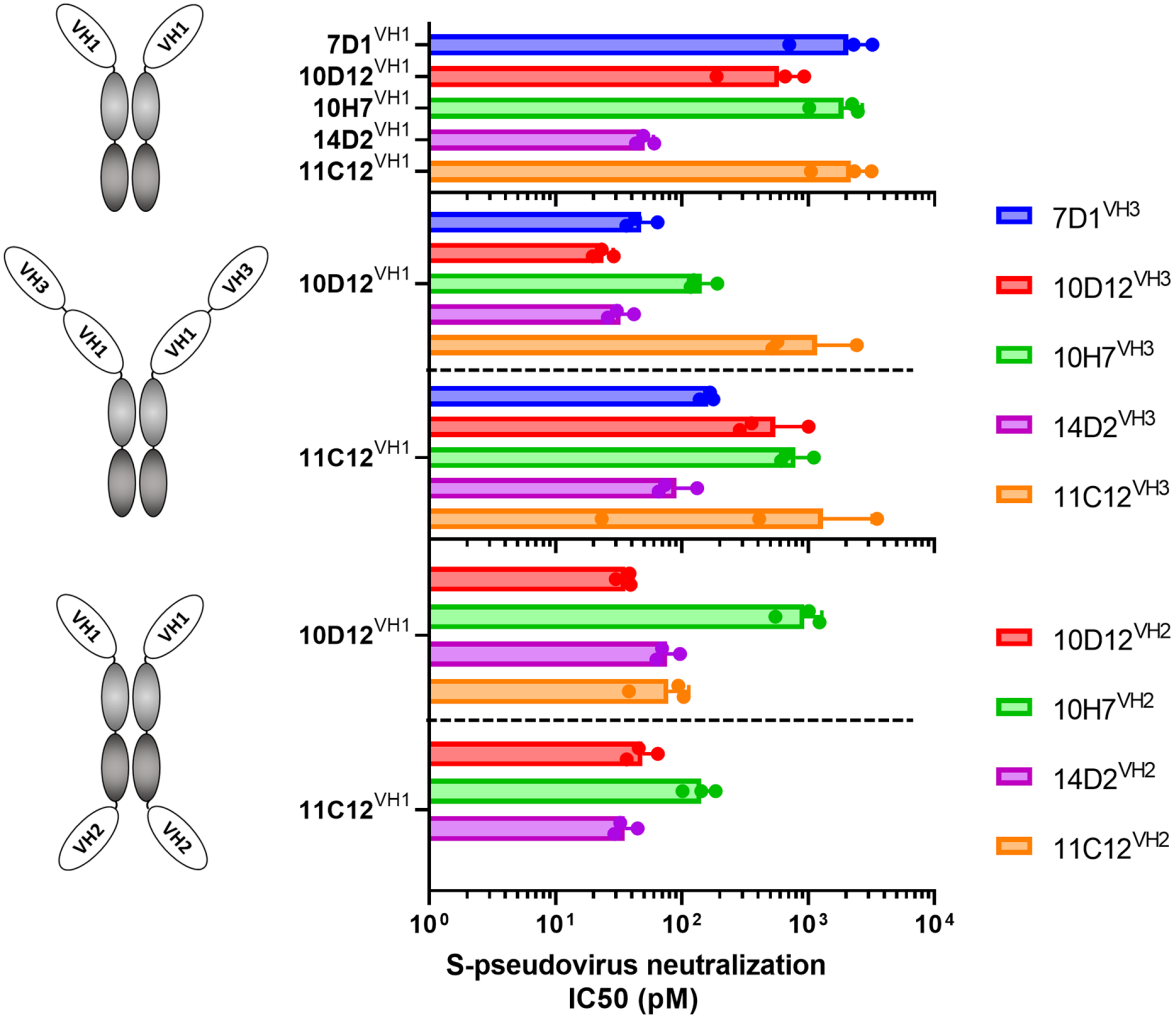
Neutralization potency of SARS-CoV-2 S-pseudotyped virus by bi- and tetravalent HCAbs. SARS-CoV-2 (Wuhan-Hu-1) pseudovirus neutralization potency (IC50 titers) on VeroE6 cells of the bivalent HCAbs (top panel), and the tetravalent HCAb designs with two VH domains at the N-terminal end of the Fc region (middle panel) or with two VH domains at opposite sides of the Fc region (low panel). Error bars indicate SD between three independent replicates, and neutralization curves are provided in **Supplementary Figure 2**.

We combined VH antigen-binding domains into two types of tetravalent antibody formats with the VH domains at three possible positions relative to the Fc region (VH1 to VH3). The VH1-VH3 format contains two VH domains in tandem (VH1 and VH3) that are positioned N-terminally of the Fc region (**Figure 2** **middle panel**). The VH1-VH2 tetravalent format harbors two VH domains (VH1 and VH2) at opposite sides of the Fc part as previously described (62, 63)(**Figure 2** **lower panel**). VH domains in the VH1-VH3 and VH1-VH2 antibody format were connected by linker regions of 25 and 14 residues in length, respectively. Ten tetravalent HCAbs in VH1-VH3 format were generated with 10D12 or 11C12 in the VH1 position and the VH3 position taken by the 7D1, 10D12, 10H7, 14D2 or 11C12 antigen-binding domain. In addition, eight tetravalent HCAbs in VH1-VH2 format were generated with 10D12 or 11C12 in the VH1 position and the VH2 position occupied by the VH domain of 10D12, 10H7, 14D2 or 11C12.

The multimerized HCAbs were tested for Wuhan-Hu-1 SARS-CoV-2 S pseudovirus neutralization and displayed half-maximal neutralization (IC50) titers ranging from 51 to 2180 pM (**Figures 2**, **S2A**). All tested tetravalent antibodies – irrespective of format and domain usage - showed lower IC50 titers relative to the monospecific bivalent counterparts, indicating that increasing the antibody valency improved virus neutralizing activity (**Figures 2B, C**). Highest neutralization potency was observed for the tetravalent antibody with 10D12 in VH1 and VH3 position (IC50: 23.9 pM), showing an ~25-fold increase in potency compared to bivalent 10D12 (*p* = 0.007, **Figure 2**; **Table S1**).

### Hexavalent mAbs retain neutralizing breadth against variants that escape bivalent parental antibodies

**Figure 3:**
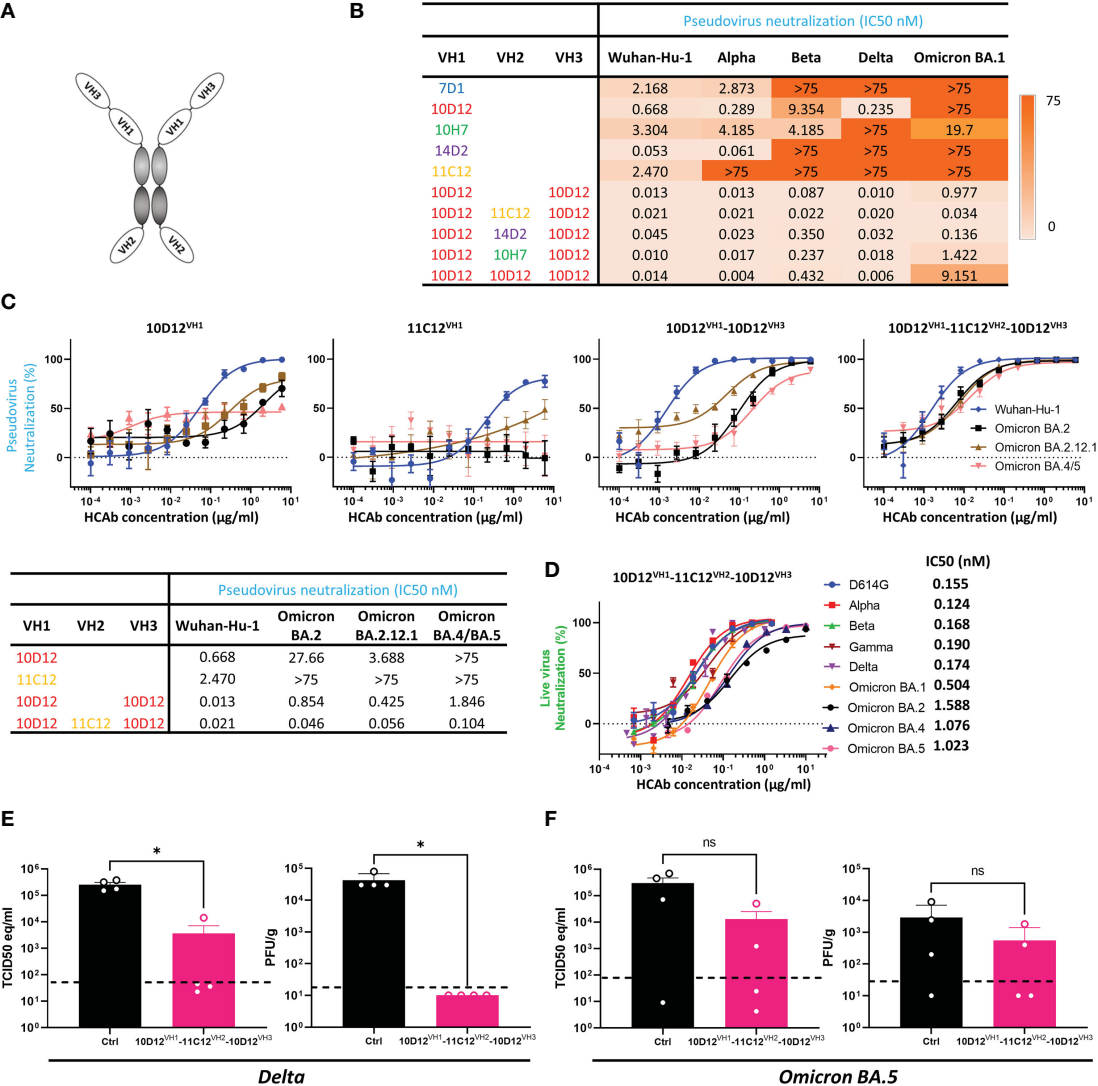
Neutralization breadth of bi-, tetra- and hexavalent HCAbs and protective activity of a hexavalent, biparatopic HCAb against Delta and Omicron BA.5 in hamsters. **(A)** Schematic depiction of hexavalent HCAb which combines three different antigen binding domains. **(B)** IC50 values of bi-, tetra- or hexavalent HCAbs against virus particles pseudotyped with S proteins of ancestral SARS-CoV-2 (Wuhan-Hu-1) and variants including Alpha, Beta, Delta and Omicron BA.1. **(C)** Bi-, tetra- and hexavalent HCAbs mediated-neutralization of viruses pseudotyped with S proteins of ancestral SARS-CoV-2 and Omicron subvariants, with calculated IC50 values displayed in the table below. **(D)** Neutralization of D614G SARS-CoV-2 and variants by 10D12^VH1^-11C12^VH2^-10D12^VH3^ HCAb. Error bars indicate SD between two independent replicates. The prophylactic efficacy of 10D12^VH1^-11C12^VH2^-10D12^VH3^ (10 mg/kg) was tested in hamsters challenged with SARS-CoV-2 Delta **(E)** or Omicron BA.5 **(F)** in comparison to non-treated (control) hamsters. Viral RNA loads (left panels) and infectious virus titers (right panels) are shown. Mann-Whitney U test was used to evaluate the statistical difference between the antibody and mock-treated groups (*, *p* < 0.05; ns, *p* > 0.05).

Based on the 10D12^VH1^-10D12^VH3^ tetravalent antibody, we further constructed hexavalent HCAbs by adding an additional antigen binding domain (11C12, 14D2, 10H7 or 10D12) at the VH2 position (**Figure 3A**). The neutralizing activity and breadth of hexavalent HCAbs and parental HCAbs were tested on Vero cells against pseudotyped SARS-CoV-2 and variants. All five parental bivalent HCAbs appeared to have lost their neutralizing activities against at least one of the VOCs, particularly towards the Omicron BA.1 variant (**Figures 3B**, **S4A**). 11C12 lost neutralization capacity against all tested variants. Increasing the valency of the HCAbs remarkably improved neutralization breadth against these variants. The tetravalent 10D12^VH1^-10D12^VH3^ efficiently neutralized Beta and Omicron BA.1 (IC50s: 0.087 and 0.977 nM, respectively), in contrast to the parental bivalent 10D12 (IC50s: 9.4 and >75 nM, respectively). It is indeed curious that 10D12VH1-10D12VH3 exhibits greater neutralization potency against Omicron BA.1 (~10-fold) compared to 10D12VH1-10D12VH2-10D12VH3. One possibility for this is that binding of the latter molecule may interfere with subsequent attachment of additional HCAb molecules (i.e., anticooperativity) (64) (**Figures 3B**, **S4B**). Among the hexavalent formats, the 10D12^VH1^-11C12^VH2^-10D12^VH3^ HCAb demonstrated the highest neutralization breadth with IC50 titers against Alpha, Beta, Delta and Omicron BA.1 VOCs reaching from 0.020 to 0.034 nM which is within 2-fold range relative to Wuhan-Hu-1 (IC50: 0.021 nM) (**Figure S3C**). Although 11C12 could neutralize none of those variants (**Figure 3B**), the addition of the 11C12 VH domain to the 10D12^VH1^-10D12^VH3^ tetravalent antibody at the VH2 position increased the neutralization potency against Omicron BA.1 by ~28 fold (*p* = 0.002) whereas neutralization potency against Wuhan-Hu-1 remained similar (*p* = 0.25). The neutralization breadth of the hexavalent 10D12^VH1^-11C12^VH2^-10D12^VH3^ was further tested against Omicron subvariants BA.2, BA.2.12.1 and BA.4 (S protein sequence identical to BA.5) in a pseudovirus assay. 10D12^VH1^-11C12^VH2^-10D12^VH3^ was able to efficiently neutralize BA.2, BA.2.12.1 and BA.4 Omicron subvariants with IC50s (0.046, 0.056 and 0.104 nM, respectively) within 5-fold range of Wuhan-Hu-1 (0.021 nM), while much lower neutralization of the three variants was seen for the parental counterparts including the 10D12 (IC50s: 27.66 [*p* = 0.02], 3.688 [*p* = 0.04] and >75 nM, respectively) and 11C12 (IC50s: all >75 nM) and tetravalent 10D12^VH1^-10D12^VH3^ (IC50s: 0.854 [*p* = 0.002], 0.425 [*p* = 0.004] and 1.846 [*p* = 0.004] nM, respectively) (**Figure 3C**). We subsequently evaluated the neutralization potency and breadth of 10D12^VH1^-11C12^VH2^-10D12^VH3^ using a live virus neutralization assay. 10D12^VH1^-11C12^VH2^-10D12^VH3^ potently neutralized an early SARS-CoV-2 strain with D614G S mutation (IC50: 0.155 nM), as well as Alpha, Beta, Gamma, Delta, and Omicron (subvariants BA.1, BA.2, BA.4 and BA.5) VOCs with IC50 titers ranging from 0.124 to 1.588 nM (**Figure 3D**). These data collectively demonstrate that engineering of antibodies with increased valency and a biparatopic design can effectively overcome neutralization resistance by SARS-CoV-2 variants against the parental antibodies.

We next evaluated the protective efficacy of 10D12^VH1^-11C12^VH2^-10D12^VH3^ against SARS-CoV-2 variants, including Delta and Omicron BA.5, in a hamster model. Syrian hamsters were administered intraperitoneally with 10D12^VH1^-11C12^VH2^-10D12^VH3^ (10 mg/kg) 24 hours before intranasal challenge with 10^4^ PFU of Delta or Omicron BA.5. 10D12^VH1^-11C12^VH2^-10D12^VH3^ treatment reduced viral RNA copies and titers in the lung of Delta-challenged hamsters at statistically significant levels compared to mock-treated hamsters (**Figure 3E**). Preventive administration of 10D12^VH1^-11C12^VH2^-10D12^VH3^ also decreased viral RNA load and infectious virus in the lung of Omicron BA.5-challenged hamsters by ~1.5 logs and ~1 log, respectively, although the difference was not statistically significant compared with mock-treated hamsters (**Figure 3F**). The lower protection by 10D12^VH1^-10D12^VH2^-10D12^VH3^ against Omicron as compared to Delta is in accordance with the lower neutralization potency seen in pseudovirus assay (17-fold) and live virus assay (6-fold) (**Figures 2B–D**). These findings demonstrate the hexavalent HCAbs can reduce viral burden *in vivo*.

### Binding sites of 10D12 and 11C12 on the SARS-CoV-2 S trimer

**Figure 4:**
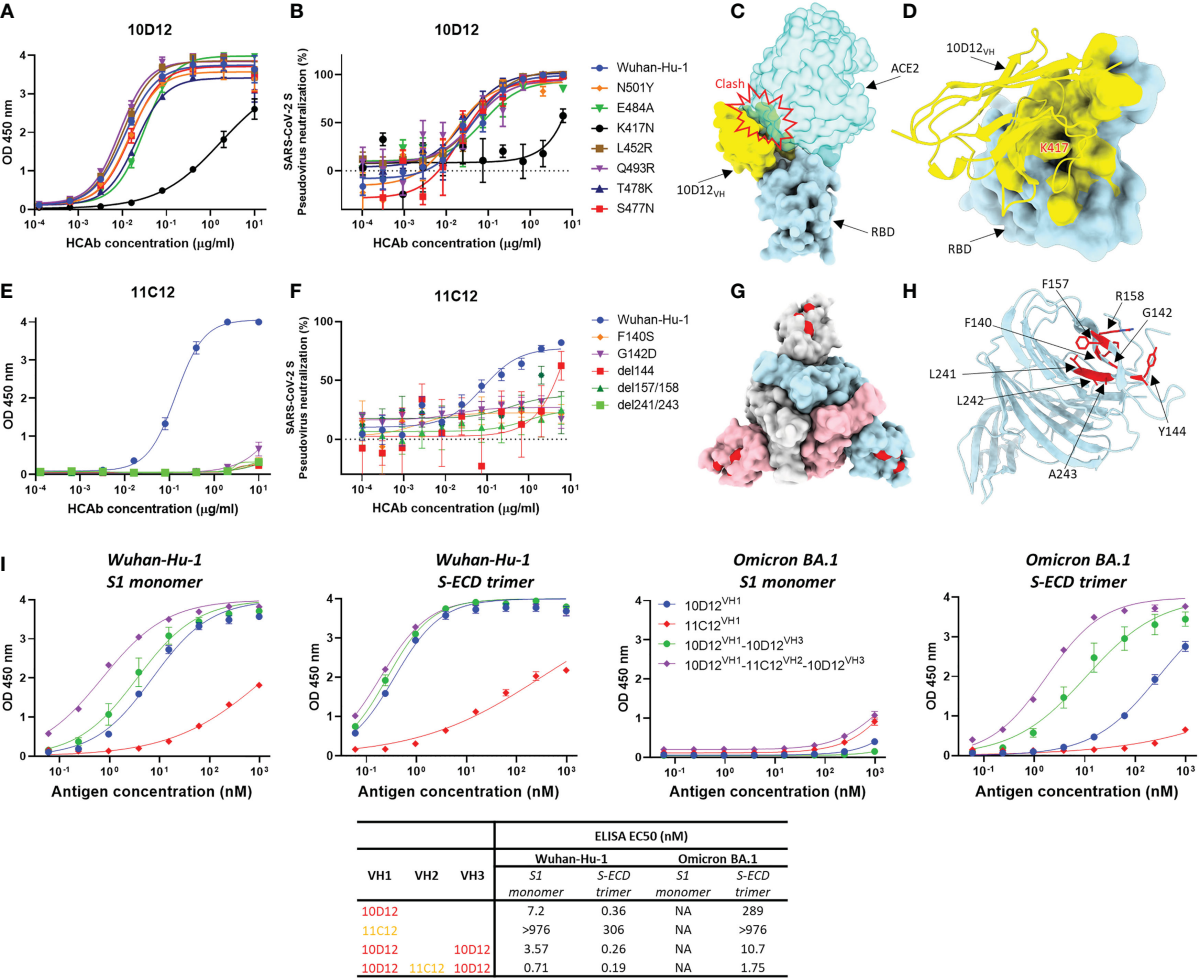
Binding sites of 10D12 and 11C12 on SARS-CoV-2 S protein trimer and ELISA binding of bi-, tetra- and hexavalent HCAbs to S antigens of ancestral and Omicron BA.1 **(A)** ELISA binding of 10D12 to ELISA plate-coated SARS-CoV-2 S1 (Wuhan-Hu-1) or S1 mutants harboring the indicated RBD mutations. **(B)** Neutralizing activity of 10D12 against pseudovirus with SARS-CoV-2 (Wuhan-Hu-1) S or S carrying indicated substitutions. **(C)** Surface representation of the 10D12-bound RBD overlaid with the RBD-bound ACE2 (PDB ID: 6M0J). **(D)** Surface representation of the SARS-CoV-2 S RBD and cartoon representation of 10D12. The 10D12 epitope residues, identified using PDBePISA, are colored yellow. **(E)** ELISA binding of 11C12 to ELISA plate-immobilized with S1 (Wuhan-Hu-1) or S1 variants carrying the indicated NTD mutations. **(F)** Neutralizing activity of 11C12 against pseudoviruses with SARS-CoV-2 (Wuhan-Hu-1) S or S variants harboring the indicated mutations. **(G)** Surface representation of SARS-CoV-2 Spike trimer (PDB ID: 6XR8) with the 11C12 binding site highlighted in red. **(H)** Close-up view showing the key residues for 11C12 binding. **(I)** Binding of monomeric S1 or trimeric S-ECD of ancestral (Wuhan-Hu-1) SARS-CoV-2 and Omicron BA.1 to plate-immobilized bivalent (10D12 and 11C12), tetravalent (10D12^VH1^-10D12^VH2^) or hexavalent (10D12^VH1^-11C12^VH2^-10D12^VH3^) HCAbs. OD 450 nm, optical density at 450 nm. Error bars indicate SD between two independent replicates.

To gain insight into the reduced neutralization of 10D12 against Beta and Omicron variants, we introduced the RBD mutations seen in the Beta and Omicron BA.1 variants to SARS-CoV-2 S1 (i.e. N501Y, E484A, K417N, L452R, Q493R, T478K and S477N) to test their effects on 10D12 binding. Substitution K417N, which is present in both variants, greatly reduced 10D12 binding to S1, while no substantial effects on binding were seen for other RBD substitutions (**Figure 4A**). The ability of 10D12 to neutralize pseudoviruses with S forms carrying these RBD mutations was also tested. Consistent with the binding data, 10D12 neutralization was compromised by the K417N mutation (**Figure 4B**), indicating K417 as a critical residue for 10D12 binding. To better understand how 10D12 interacts with the RBD, we performed cryo-electron microscopy (cryo-EM) analysis on the 6P-stabilized Wuhan-Hu-1 S-ECD in complex with the bivalent 10D12 HCAb. Two distinct conformations of the S-ECD, with either two or three RBDs in the open conformation, were obtained following 3D classification (**Figure S6**). Density consistent with the 10D12 heavy chain variable region was observable on each open RBD. Subsequent 3D refinement of the fully and partially open S-ECD conformations produced density maps with global resolution estimates of 3.1 Å and 3.3 Å, respectively (**Figures S7A–D, G–H**). Because of the considerable flexibility of the open RBD, the epitope-paratope region was poorly resolved. To improve the interpretation of the 10D12 binding site, focused refinement was performed on the RBD-HCAb interface. The resulting 4.1 Å resolution locally refined map did not allow interpretation at the level of side chains but did facilitate fitting of a 10D12 AlphaFold model and RBD crystal structure into the EM density (**Figures S7E, F, I**). Consistent with our solid-phase data, 10D12 is a class 1 antibody with an epitope that overlaps with the ACE2 binding site, preventing receptor engagement through steric hindrance (**Figure 4C**). Based on our model, the 10D12 epitope appears to comprise RBD residues 403, 408, 415-417, 420-421, 453, 455-460, 473-477, 486-487, 489, 493, 495 and 505. In line with our mutagenesis experiments, K417 seems to be a key epitope residue (**Figure 4D**). HCAb 10D12 uses each of its CDR loops to engage the RBD, with the paratope comprising residues 2, 26-28, 30-34, 52-58, 97-103 and 108-109. Sequence comparison to other identified anti-SARS-CoV-2 antibodies revealed that 10D12 shares 87.5% sequence homology with the heavy chain variable region of C1A-C2 (**Figure S8A**). These molecules share the same IGHV-53 germline and structural comparison reveals that the orientations of the RBD-bound VH domains are highly similar (**Figures S8B–D**), with the aligned complexes deviating by a root mean square deviation (RMSD) value of 1.78 Å across 313 Cα atoms pairs. Like C1A-C2, HCAb 10D12 can only bind to the open RBD as it would clash with the adjacent S protomer in the context of the closed spike. However, it is worth noting that the interface between 10D12 and the RBD (~720 Å) is considerably smaller than that of C1A-C2 (~1370 Å), because the latter uses both its heavy and light chain to bind to the spike protein. To understand HCAb 10D12 binding in the context of the spike trimer, we fitted our RBD-VH model into the cryo-EM map of the S-ECD with two open RBDs. The orientation of the two bound VH domains, and the short distance between their C-termini, would be compatible with bivalent binding of HCAb 10D12 to the spike (**Figure S9A**). Similarly, the distance between the C-terminus and N-terminus of adjacent RBD-bound VH domains, in the context of the fully open S-ECD, could be bridged by the long linkers used to connect the VH3 and VH1 positions in our most potent hexavalent construct (**Figure S9B**).

Efforts to obtain the cryo-EM structure of the S trimer in complex with 11C12 were not successful, but site-directed mutagenesis scanning allowed us to pinpoint the key residues on S for 11C12 binding. Mutations in Alpha, Beta, Delta and Omicron variants are expected to reduce binding by 11C12 as these variants cannot be neutralized. To identify the 11C12 epitope, NTD mutations in these variants that are found in or close to the antigenic supersite loops were introduced in SARS-CoV-2 S1 or full-length S to assess impact on 11C12 binding and neutralization, respectively. Substitution F140S was included as an escape mutation for NTD-directed neutralizing antibodies (65). G142D (seen in Delta and Omicron BA.1), Δ144 (Alpha), Δ157/158 (Delta) and Δ241-243 (Beta) all abrogated binding to 11C12. These data map the 11C12 epitope to the NTD antigenic supersite and explain neutralization resistance of these variants by 11C12, indicating an important role of these residues in S binding. A F140S resistance mutation against NTD-targeting neutralizing monoclonal antibodies included in this analysis also conferred binding loss to 11C12 (**Figure 4E**). In agreement with the binding data, 11C12 lost neutralization of pseudoviruses with S variants carrying these mutations (**Figure 4F**). Thus, the mutations that impact 11C12 binding and neutralization map to the NTD antigenic supersite recognized by potent neutralizing monoclonal antibodies (**Figures 4G, H**) (8), indicating that 11C12 targets the same region.

To mechanistically understand the enhanced neutralization potential and breadth upon antibody multimerization, we assessed the binding potency and breadth for the hexavalent 10D12^VH1^-11C12^VH2^-10D12^VH3^ relative to its bivalent (10D12^VH1^ and 11C12^VH1^) and tetravalent (10D12^VH1^-10D12^VH3^) counterparts. We coated an equimolar amount of each of the antibodies to the ELISA plate and determined their binding capacity to serially diluted monomeric S1 or trimeric S-ECD of the ancestral SARS-CoV-2 (Wuhan-Hu-1) and Omicron BA.1 variant. Overall, the hexavalent HCAb showed the highest binding avidity to both S antigens compared to the bi- and tetravalent counterparts (**Figure 4I**). Binding to trimeric S-ECD was higher for all HCAbs compared to monomeric S1 binding, indicating that increase in multivalency enhances binding to multimeric S antigens. No apparent binding to monomeric BA.1 S1 was observed for all HCAbs, congruent with the absence of binding of 11C12 and 10D12 to S1 variants containing BA.1 escape mutations (**Figure 4A**). In contrast to 11C12, low but detectable binding was seen of the bivalent 10D12 to the trimeric BA.1 S-ECD. Increased binding to BA.1 S-ECD trimer was observed for the tetravalent 10D12^VH1^-10D12^VH3^ whereas the highest binding was seen for the hexavalent 10D12^VH1^-11C12^VH2^-10D12^VH3^. EC50 titer of the latter was only 3-fold higher relative to binding to the ancestral Wuhan-Hu-1 S-ECD trimer. The 11C12 antigen-binding domain contributed to the overall avidity of the hexavalent antibody as its EC50 titer was 6-fold lower compared to the tetravalent 10D12^VH1^-10D12^VH3^. We subsequently analyzed binding kinetics of the HCAbs to these S antigens by BLI. Consistent with the ELISA data, the BLI data demonstrate that enhancing the valency of the HCAbs effectively increases overall avidity to trimeric S-ECD - but not to monomeric S1 - of the BA.1 variant (**Figure S5**). This phenomenon may be explained by an increased numbers of binding events or the increase in local concentration of binding moieties.

## Discussion

The ongoing evolution of Omicron has resulted in accumulation of mutations in S epitopes rendering all currently authorized monoclonal antibody therapies ineffective. New therapeutic antibodies with broad-spectrum activity are thus urgently needed. Antibodies with extraordinary binding breadth have been identified targeting conserved epitopes in the S2 fusion subunit. However, these antibodies exhibit limited virus neutralization potency questioning their potency to be used as therapeutics (66–70). Hence, alternative antibody design strategies that increase neutralization potency and breadth are required to fight emerging SARS-CoV-2 variants. One promising strategy to counteract variant escape is the development of multivalent and multispecific antibodies using single-domain antibodies that can be easily designed through molecular linkage into multimeric forms with extra binding properties (33).

Multimerization of single-domain antibodies and other therapeutic agents can substantially improve virus neutralization potency through avidity effects or improved intra- and inter-spike crosslinking, as has been shown for SARS-CoV-2 and other respiratory viruses (21–24, 26–32, 35–39, 41–43, 71–73). Generation of multidomain antibodies targeting multiple epitopes may also enhance neutralization breadth. Recent reports have shown that avidity engineering of sdAbs – including the generation of multi-paratopic designs – can lead to a considerable increase in neutralization breadth that includes Omicron variants (BA.1 and BA.2) (21, 22). In our study, we confirmed and extended the concept of avidity engineering of sdAbs to generate antibodies with extraordinary neutralization breadth. Our hexavalent 10D12^VH1^-11C12^VH2^-10D12^VH3^ could neutralize all the SARS-CoV-2 variants tested including Omicron BA.1, BA.2, BA.2.12.1 and BA.4/BA.5. Remarkably, its neutralization breadth was not achieved by binding to conserved epitopes since the monospecific bivalent counterparts 10D12 and 11C12 HCAbs had lost their neutralizing activity towards Omicron variants by single-site mutations which drastically reduced their spike binding affinity to low (10D12) or undetectable (11C12) levels. Nevertheless, tethering of 10D12 and 11C12 into a hexavalent antibody notably restored the binding and neutralization to Omicron. Binding restoration was only seen to the trimeric S ectodomain and not to the monomeric S1. How the apparent affinity of multivalent target recognition can increase so notably is not fully resolved. The spatial arrangement of the 10D12 and 11C12 epitopes in the S trimer may allow simultaneous binding of the epitope-binding regions, leading to avidity effects (72). Alternatively, binding of one antigen-binding domain to its epitope on the trimer may bring the other binding domains close to their target, increasing the local concentration and probability of an interaction with another site. Hence the tethered nature of the antigen-binding domains results in large increases in ‘target residence time’, which can lead to rapid rebinding by other domains upon dissociation of a single antigen-binding domain (74). The long linkers (14 or 25 residues) that are used to connect antigen-binding domains increase the conformational flexibility needed in both scenarios. Despite the lack of apparent spike binding by bivalent 11C12 HCAb to trimeric BA.1 spike, addition of two 11C12 VH domains to the tetravalent 10D12^VH1^-10D12^VH3^ at the VH2 position improved binding to the BA.1 S trimer and BA.1 neutralization, indicating that the preserved 11C12 epitope residues in BA.1 S can still contribute to the overall binding affinity of the hexavalent antibody. Considering our structural and functional data, we propose a tentative model in which 10D12^VH1^-10D12^VH3^ simultaneously engage the RBDs within a spike trimer, leaving an additional 10D12^VH3^ to participate in inter-spike binding. Due to the long length of the linkers used in our construct, we believe that the two 11C12^VH2^ molecules, present on the C-terminus of the molecule, could engage in inter- or intra-spike binding and thus contribute to the neutralization potency of our hexavalent molecule (**Figure S9C**).

The hexavalent antibody format used with multiple human VH domains linked to different sites of the Fc region of a human IgG1 antibody combines several features with potential therapeutic benefits. Inclusion of the Fc region confers avidity through Fc homodimerization. In addition, Fc-containing antibodies are likely to have a prolonged serum half-life through interaction with the neonatal Fc receptor (75). Moreover, Fc addition may enable recruitment of Fc-mediated effector functions that was shown to enhance *in vivo* therapeutic efficacy of SARS-CoV-2 monoclonal antibodies (76–79). Furthermore, our strategy to add on VH domains to opposite site of the Fc fragment may facilitate multivalent binding across longer distances and thereby increase the likelihood of inter-spike cross-linking. Lastly, the fully human nature of the multimerized antibodies relative to camelid derived antibodies may reduce the risk of immunogenicity and antidrug antibody (ADA) formation in humans, particularly upon repeated antibody administrations (80).

Collectively our work provides further evidence that the tethered design of single-domain antibodies can mitigate neutralization escape of antigenically drifted SARS-CoV-2 variants (21, 22). Engineering antibodies with increased valency and the potential to target multiple paratopes is hence a promising avenue to develop potent and broad-spectrum antibodies towards SARS-CoV-2 and variants.

## Data availability statement

The data presented in the study are deposited in the Figshare repository withe the link shown below: https://figshare.com/articles/dataset/HCAb/22006313. The globally and locally refined cryo-EM maps have been deposited to the Electron Microscopy Data Bank under the accession codes EMD-16480, EMD-16481 and EMD-16490. The pseudoatomic model of the 10D12-bound RBD has been deposited to the PDB under the accession code 8C8P. Nucleotide sequences of the variable regions of the heavy-chain only antibodies 10D12, 10H7, 7D1, 11C12 and 14D2 are available from GenBank under the following accession numbers OP903629, OP903630, OP903631, OP903632 and OP903633, respectively.

## Ethics statement

The animal study was reviewed and approved by Institutional Animal Welfare Body, Erasmus MC.

## Author contributions

Experiment design: WD, FG, DH, and B-JB; gene cloning, protein expression and purification: WD, JL; isolation and characterization of antibodies: WD, RJ, RH and DD; binding and neutralization assays, WD, AM, MC, JL and MD; hamster experiment, MR; cryo-EM data collection, processing, and model building, IM and DH.; data analysis, WD, DH and B-JB.; supervision, BH and B-JB; study conception and coordination, B-JB; manuscript writing, WD and B-JB, with input from all other authors. All authors contributed to the article and approved the submitted version.
