# Supplementary material for: Avidity engineering of human heavy-chain-only antibodies mitigates neutralization resistance of SARS-CoV-2 variants
Source: Front Immunol. 2023 Feb 21;14:1111385. doi: 10.3389/fimmu.2023.1111385 (PMC9990171; doi:10.3389/fimmu.2023.1111385)
Supplement: Supplementary file 1 [file DataSheet_1.docx]

Supplementary Material


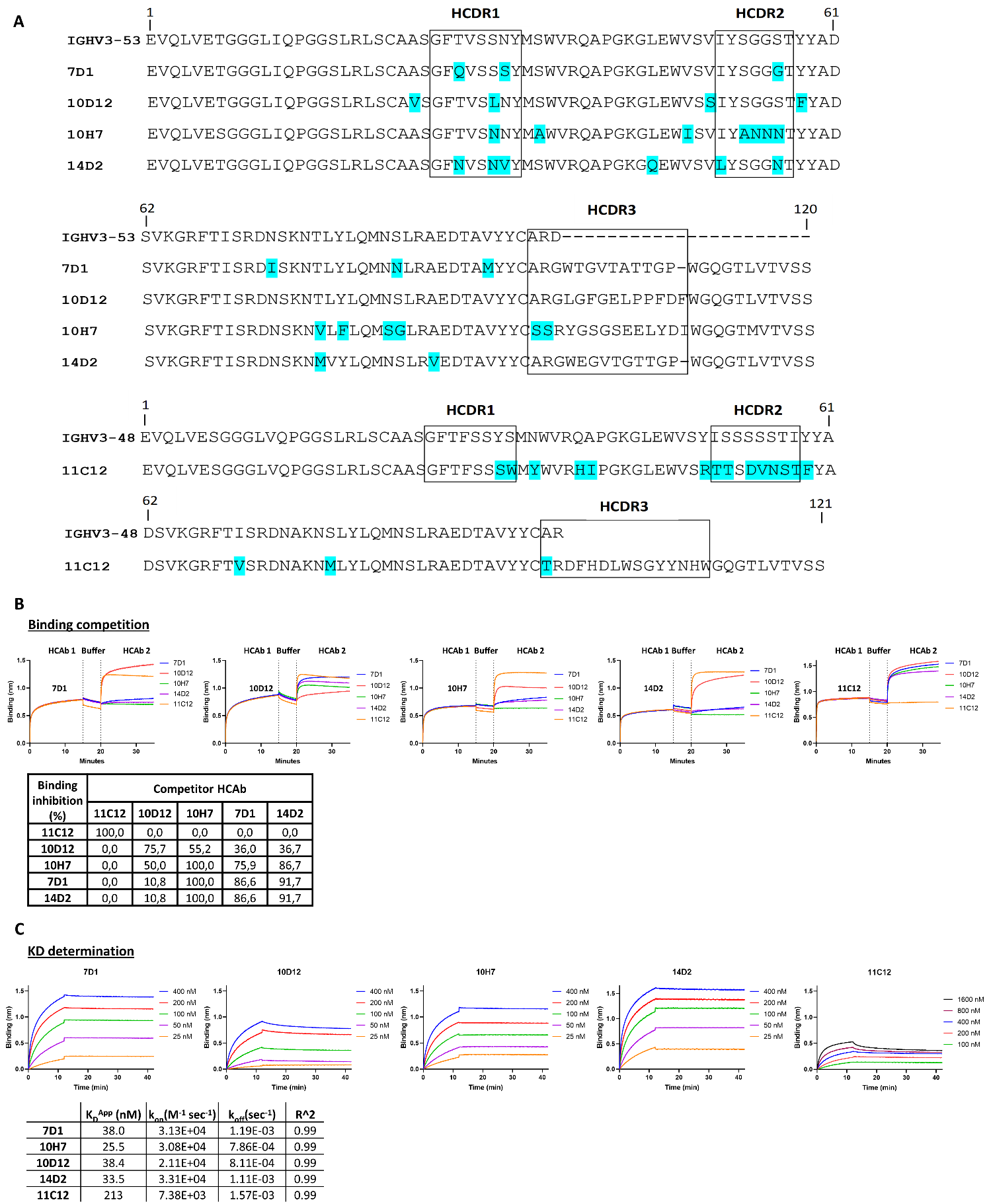


**Fig.S1** (A) Alignment of variable domain sequences of HCAb antibodies with the germline sequence. Somatic hypermutations in the antibody sequences are highlighted in green, and the three complementarity determining regions (CDRs) are boxed. (B) For epitope binning the HCAbs were tested pairwise for competitive binding to the soluble trimeric SARS-CoV-2 S ectodomain (S-ECD) using biolayer interferometry (BLI). The strep-tagged SARS-CoV-2 S-ECD antigen was loaded to the Protein A biosensor via a Strep-tag MAb. S-ECD antigen on the biosensor was exposed to binding with a first HCAb (HCAb 1) and subsequently to binding by a second competitor HCAb (HCAb 2), with a 5 min washing step in between. (C) Binding kinetics of HCAb to SARS-CoV-2 S-ECD trimer measured by BLI. The HCAb immobilized onto the Protein A sensor was exposed to twofold serially diluted SARS-CoV-2 S-ECD, followed by a dissociation step in PBS. K_D_^App^ reflects the “apparent affinity” between HCAb and S-ECD trimer; k_on_ : association rate constant, k_off_ : dissociation rate constant.


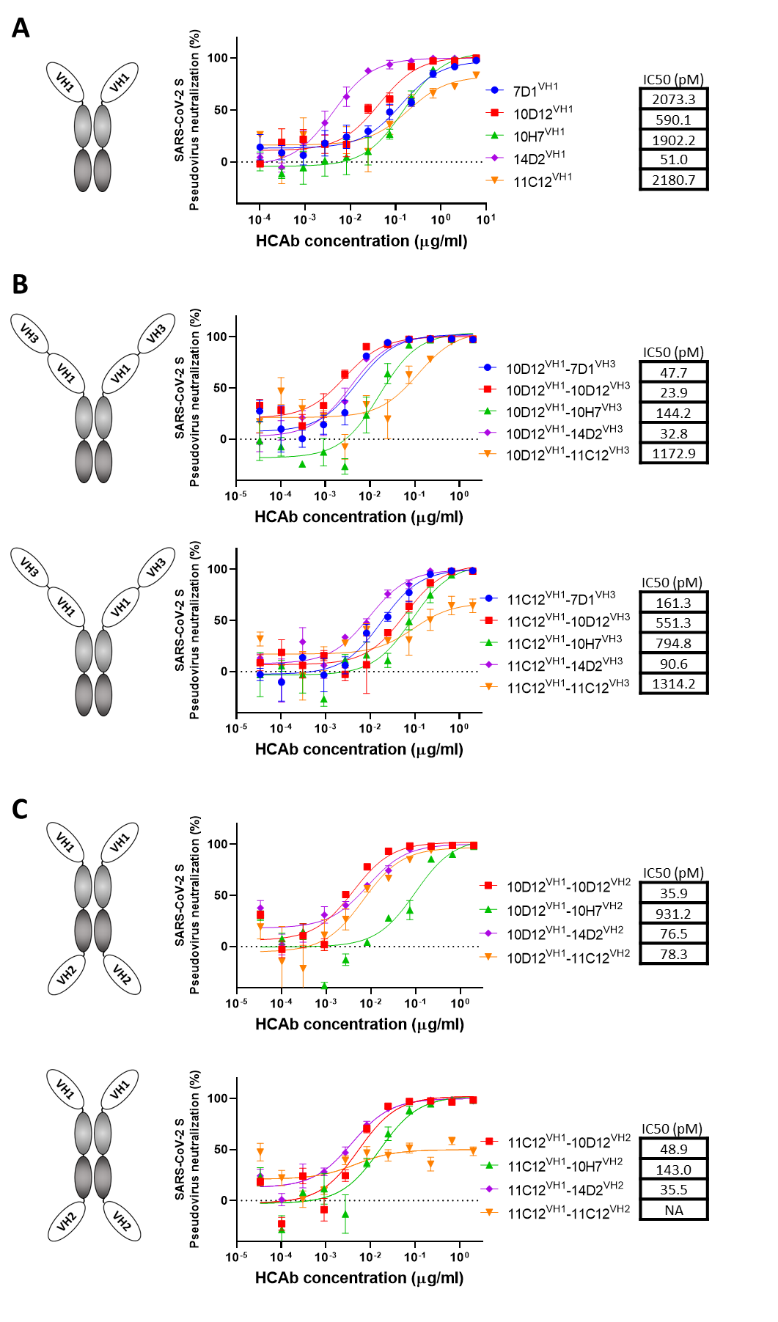


**Fig.S2** Neutralization potency of bivalent (A) and tetravalent (B and C) heavy chain antibodies against SARS-CoV-2 S pseudovirus (Wuhan-Hu-1) on Vero-E6 cells. Error bars indicate standard deviation between three independent replicates. IC50 values are indicated. NA: not applicable. Related to Fig 2.


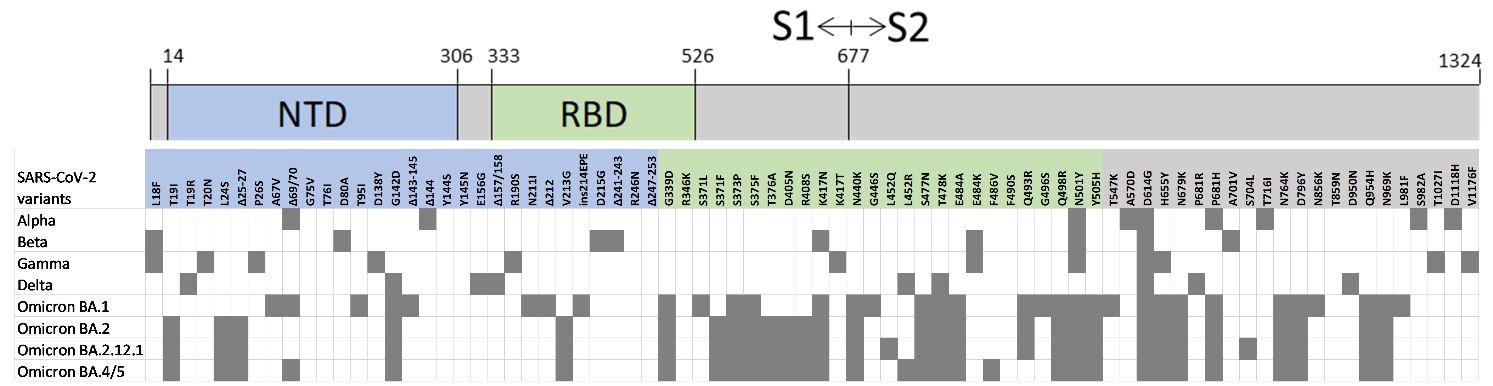


**Fig.S3** Schematic representation of S protein mutations found in SARS-CoV-2 variants, relative to ancestral SARS-CoV-2. The S protein N-terminal domain (NTD; in blue), receptor binding domain (RBD; in light green) and the S1/S2 junction are indicated.


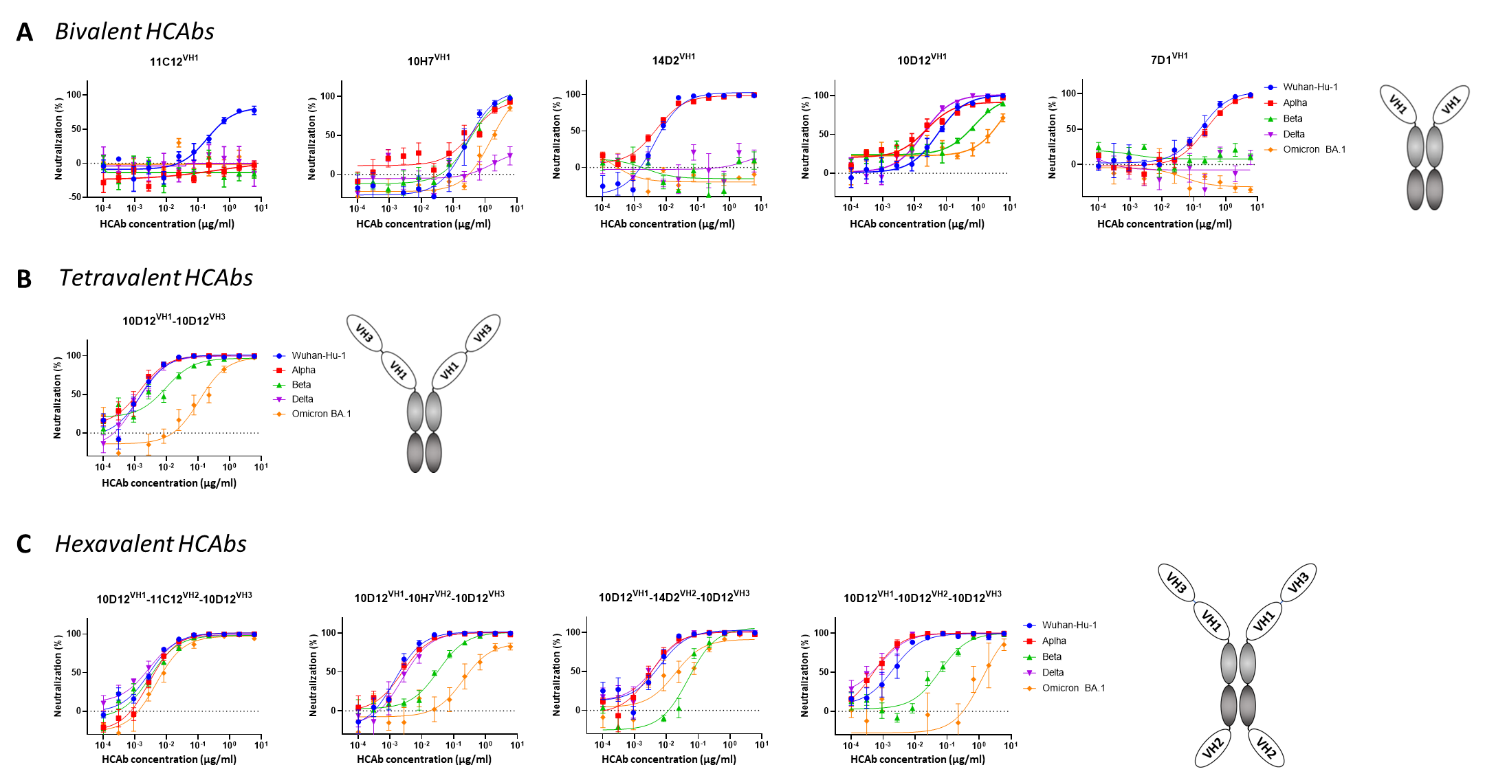


**Fig.S4** Neutralization curves for bivalent (A), tetravalent (B) and hexavalent (C) heavy chain antibodies of viruses pseudotyped with S proteins of the indicated SARS-CoV-2 variants. Related to Fig.3B.


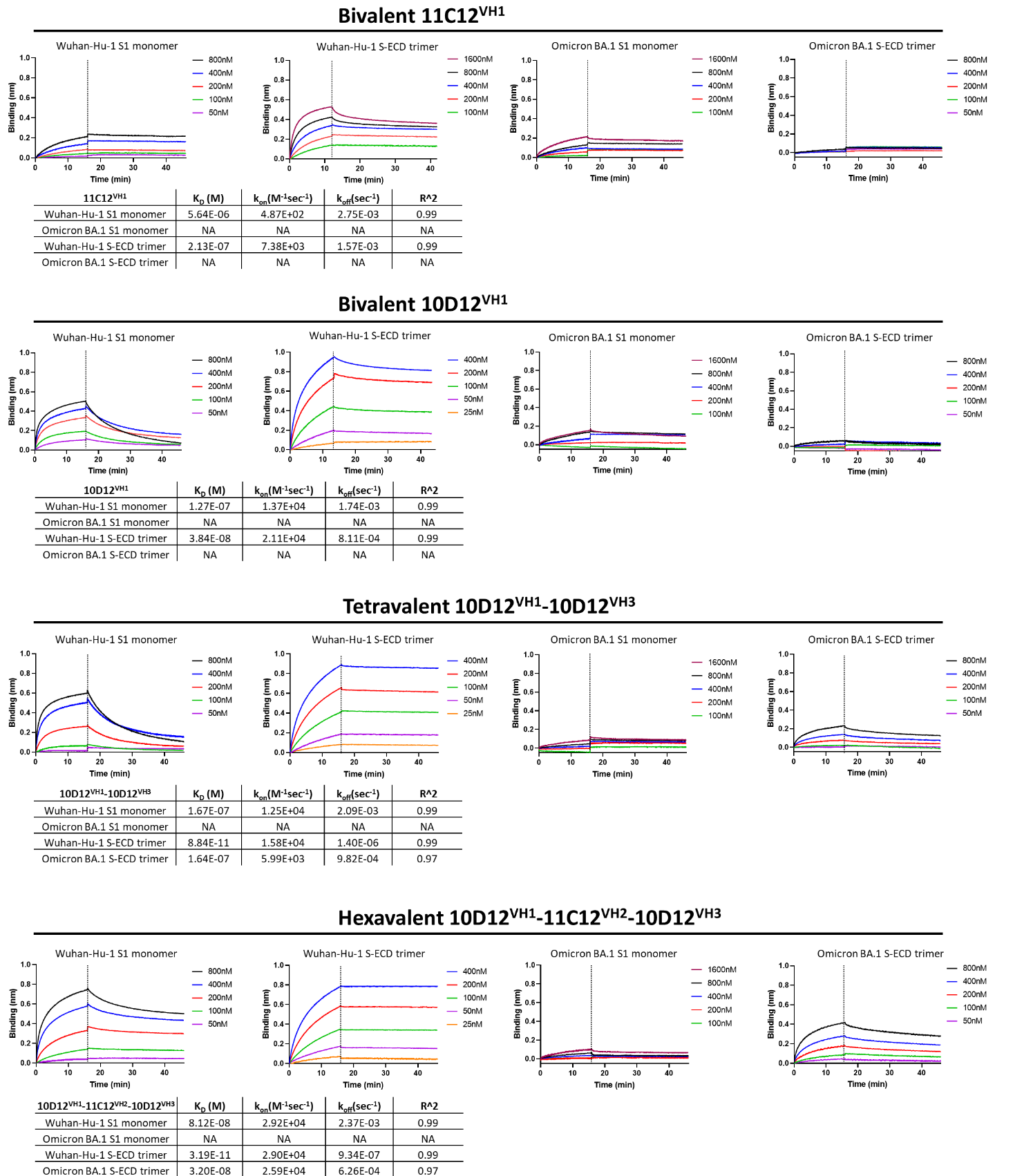


**Fig.S5** Binding kinetics of HCAbs against monomeric S1 or trimeric S proteins of ancestral SARS-CoV-2 or of Omicron BA.1. Binding kinetics of HCAbs to SARS-CoV-2 S was measured by biolayer interferometry (BLI). HCAbs immobilized onto the Protein A biosensors were exposed to twofold serially diluted recombinant S1 monomer or S ectodomain trimer of Wuhan-Hu-01 or Omicron BA.1, followed by a dissociation step in PBS. KD: equilibrium dissociation constant. k_on_: association rate constant, k_off_: dissociation rate constant. NA (not applicable): low binding affinity prohibits calculation of binding kinetic parameters.


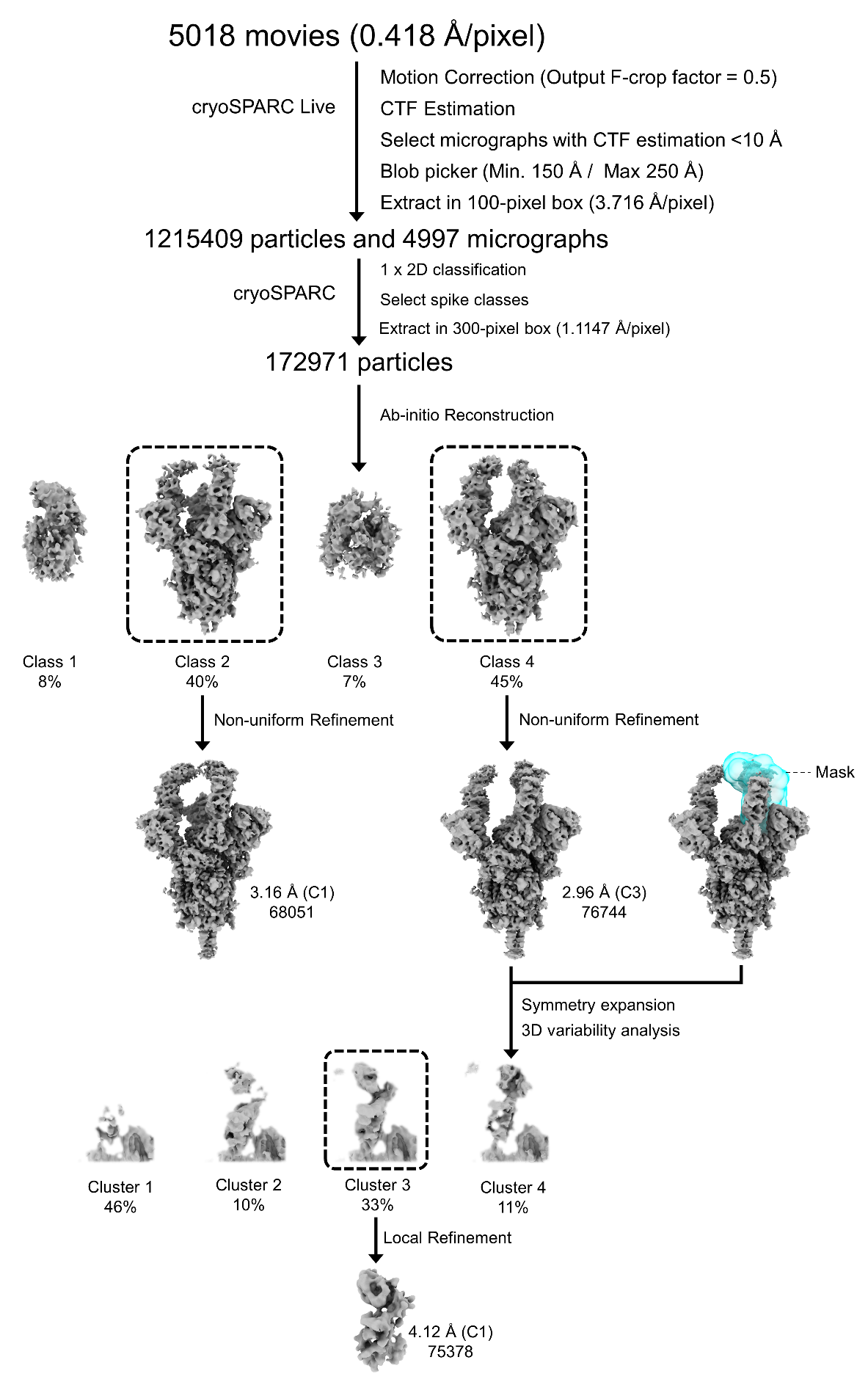


**Fig.S6** Cryo-EM data processing pipeline for the SARS-CoV-2 S ectodomain in complex with HCAb 10D12


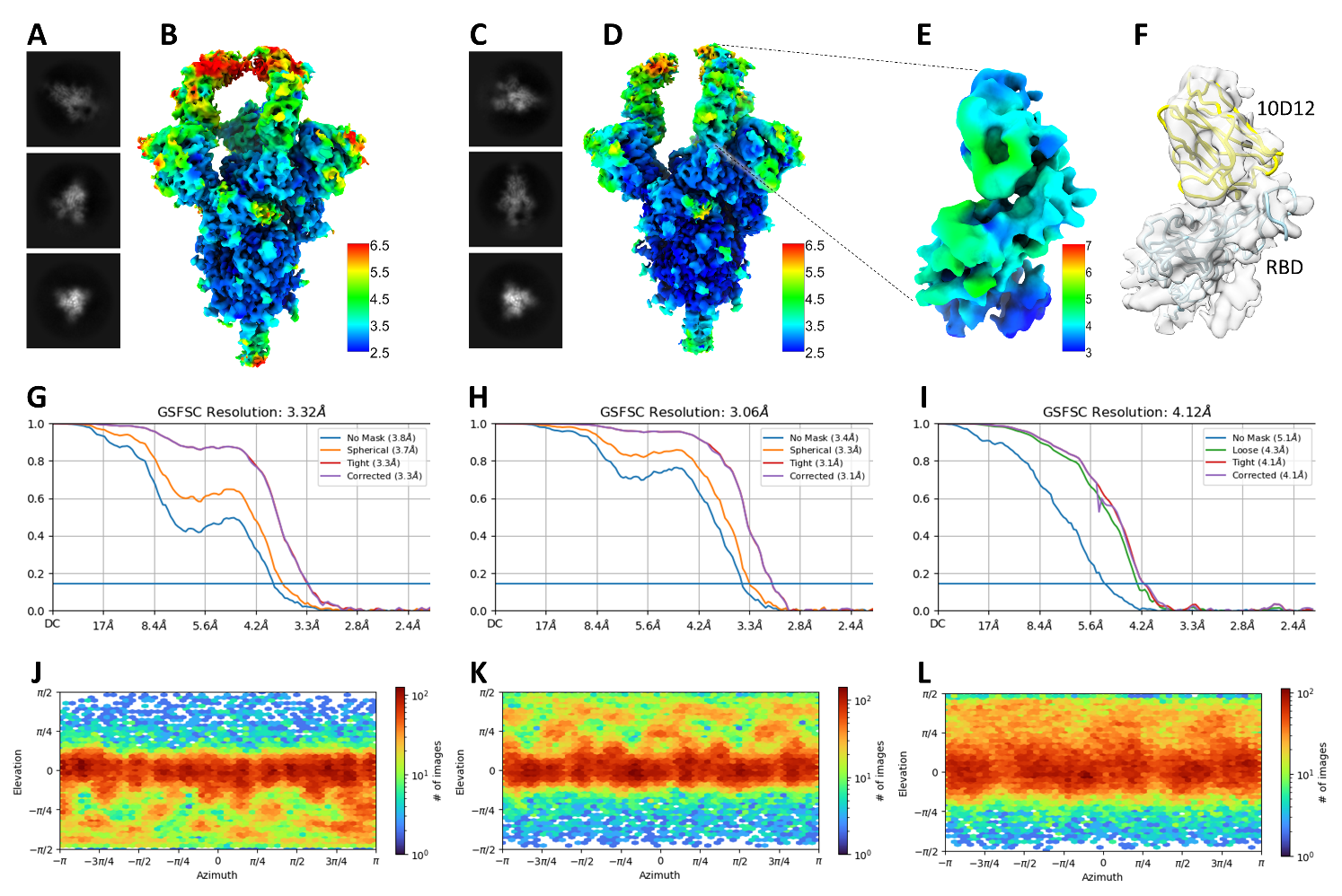


**Fig.S7** Cryo-EM data processing of SARS-CoV-2 S bound to 10D12. (A) Representative reference-free 2D class averages of the partially open S-ECD in complex with 10D12, generated in cryoSPARC. (B) Cryo-EM density map for the globally refined spike-10D12 complex colored according to local resolution (Å). (C) As shown in A, for the fully open S-ECD in complex with 10D12 (D) As shown in B, for the fully open S-ECD in complex with 10D12. (E) Cryo-EM density map for the locally refined RBD-10D12_VH_ complex, colored according to local resolution (Å). (F) Cryo-EM density for the locally refined epitope-paratope region with the fitted atomic coordinates shown. The RBD is colored blue and 10D12_VH_ is colored yellow. (G-H) Gold standard Fourier shell correlation (FSC) curves generated from the independent half maps of the globally refined maps, and (I) for the locally refined map. (J-K) Angular distribution calculated in cryoSPARC for particle projections in the global refinements and (L) local refinement.


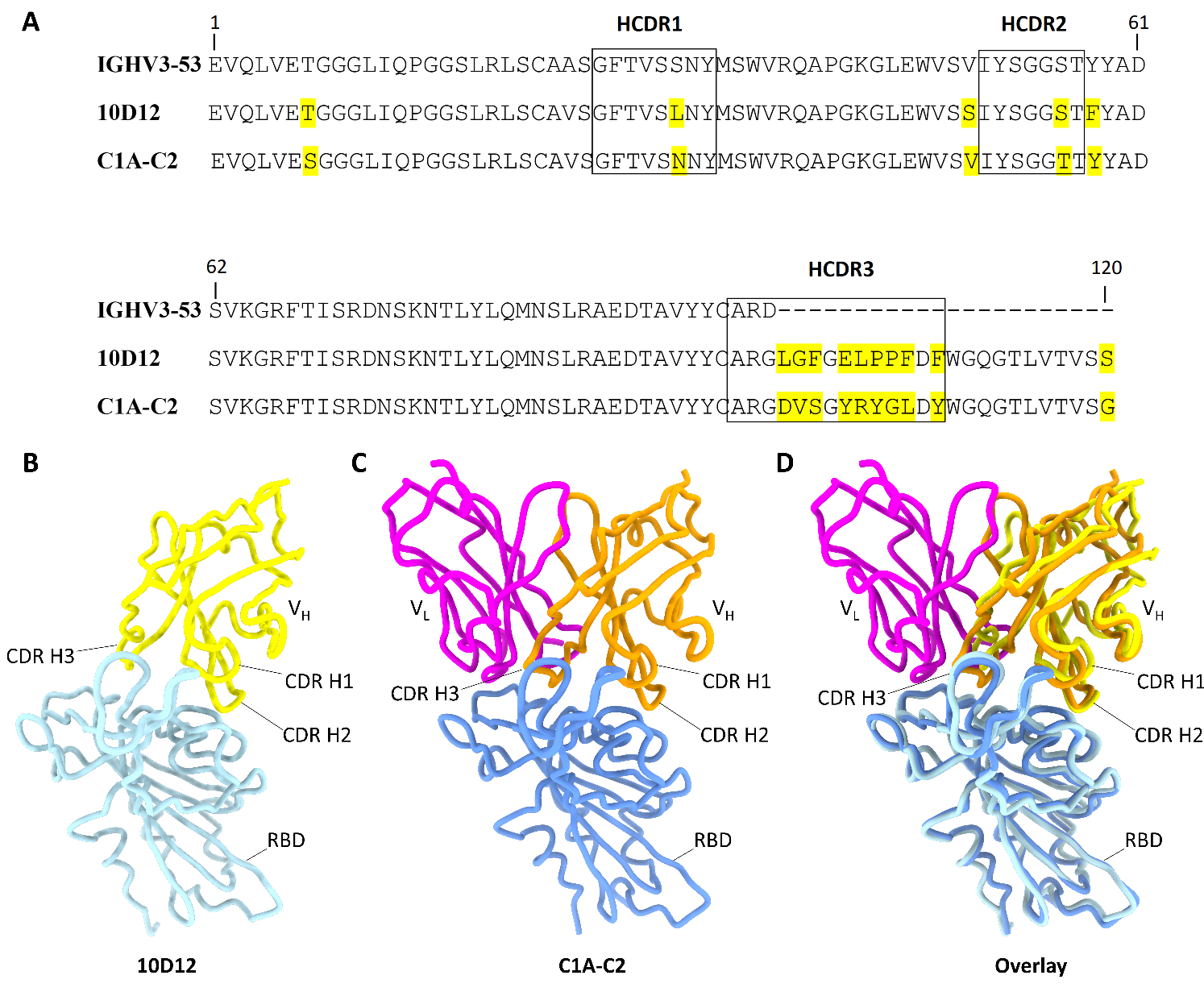


**Fig.S8 (**A) Heavy chain variable region sequence alignment of 10D12 with another RBD-binding mAb C1A-C2 that shares the same germline IGHV3-53. Differences between antibody sequences are highlighted in yellow. The CDRs are boxed. Side-by-side comparison of SARS-CoV-2 RBD bound by 10D12 (B), C1A-C2 (PDB ID: 7KFX) (C) and the overlay of those two structures (D).

**
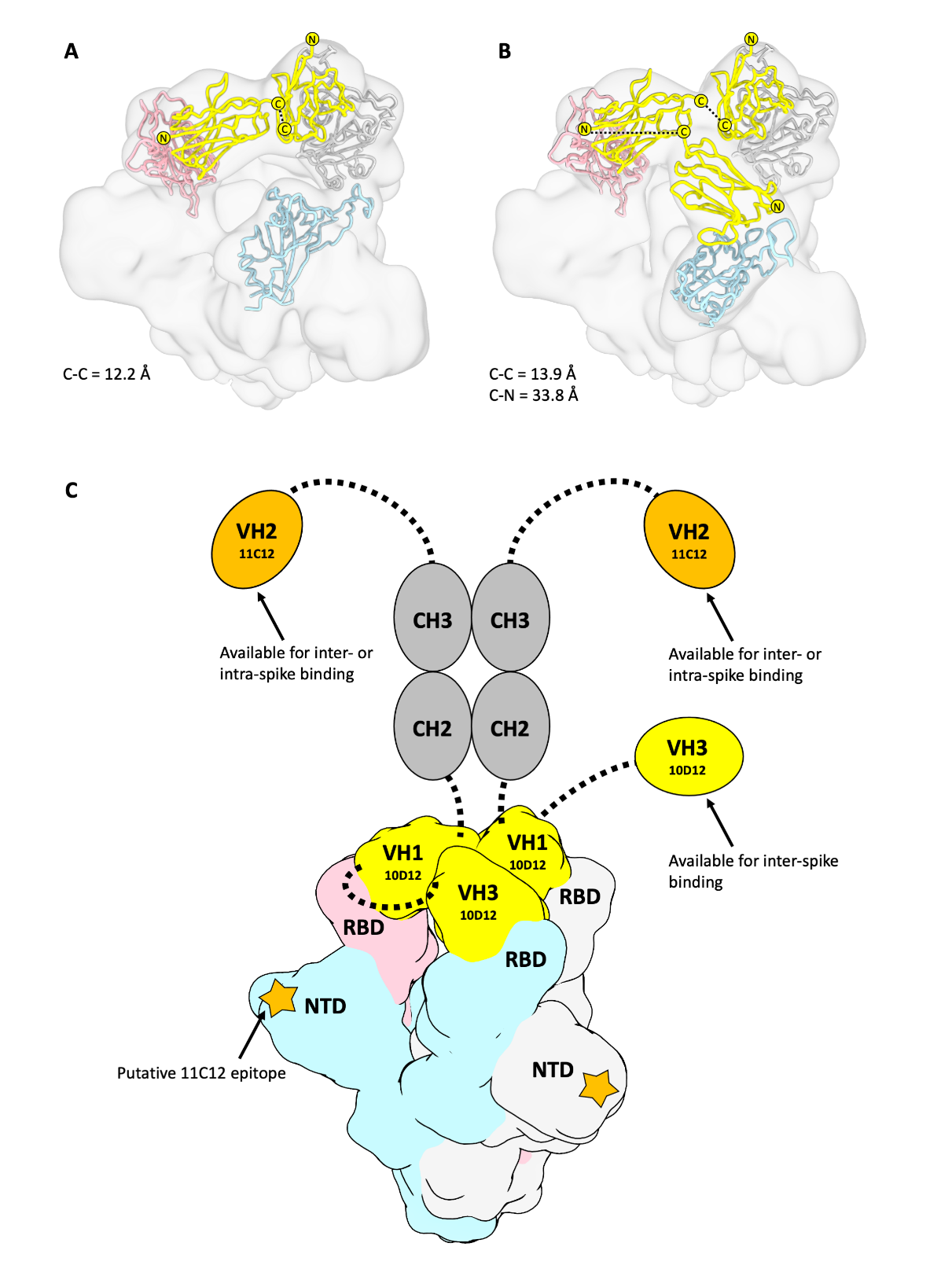
**

**Fig.S9** (A) Gaussian filtered EM density map for the partially open trimeric SARS-CoV-2 S-ECD with two copies of the RBD-10D12 VH model (yellow ribbon), and one unbound RBD, fitted as a rigid bodies. The spike protomers are colored blue, grey and pink. (B) As shown in A for the fully open S-ECD reconstruction. The N- and C-termini of the bound VH domains and distances between their termini - indicated by dashed lines - are shown. (C) Proposed model for 10D12^VH1^-11C12^VH2^-10D12^VH3^ binding to the trimeric spike, with three out of four 10D12 domains bound to the trimeric spike and remaining VH domains available for inter- and/or intra-spike binding.

**Table S1** Summary of cryo-EM data acquisition, image processing and model refinement statistics

| **Data Collection** |  |  |  |
| --- | --- | --- | --- |
|  |  |  |  |
| Microscope |  | Titan Krios |  |
| Voltage (keV) |  | 300 |  |
| Nominal magnification |  | 105,000x |  |
| Movie acquisition rate |  | ~231 per hour |  |
| Detector |  | K3 |  |
| Energy filer |  | BioQuantum |  |
| Slit width (eV) |  | 20 |  |
| Calibrated pixel size (Å) |  | 0.418 |  |
| Cumulative exposure (e/Å^2^) |  | 50 |  |
| Dose rate (e/pixel/sec) |  | 21 |  |
| Underfocus range (μm) |  | 0.6 to 2.4 |  |
| Micrographs collected |  | 5018 |  |
|  |  |  |  |
| **Reconstruction** | EMD-16481 | EMD-16480 | EMD-16490 |
| Final particles (no.) | 68.051 | 76.744 | 75.378 |
| Symmetry | C1 | C3 | C1 |
| B-factor (Å^2^) | -74 | -92 | -181 |
| **Resolution (Å)** |  |  |  |
| FSC 0.5 (masked) | 3.7 | 3.3 | 5.1 |
| FSC 0.143 (masked) | 3.3 | 3.1 | 4.1 |
| Resolution range (local) | 2.4-31.6 | 2.5-8.9 | 3.2-17.3 |
|  |  |  |  |
| **Refinement (local)** | **-** | **-** | PDB 8C8P |
| Protein residues/atoms | **-** | **-** | 314/2451 |
| N-glycans/atoms | **-** | **-** | 0 |
| **Resolution (Å)** |  |  |  |
| FSC 0.5 | **-** | **-** | 6.3 |
| **Map correlation coefficient** |  |  |  |
| Mask | **-** | **-** | 0.69 |
| Box | **-** | **-** | 0.63 |
| Volume | **-** | **-** | 0.71 |
| Peaks | **-** | **-** | 0.30 |
| **R.M.S. deviations** |  |  |  |
| Bond Lengths (Å) | **-** | **-** | 0.002 |
| Bond Angles (°) | **-** | **-** | 0.669 |
| **MolProbity** |  |  |  |
| Overall score | **-** | **-** | 1.43 |
| Clashscore | **-** | **-** | 6.69 |
| Ramachandran outliers (%) | **-** | **-** | 0 |
| Ramachandran favoured (%) | **-** | **-** | 97.74 |
| Rotamer outliers (%) | **-** | **-** | 0 |
| C-beta outliers | **-** | **-** | 0 |

**Table S2** Statistical comparison of IC50 values of bi- and tetravalent HCAbs against SARS-CoV-2 S pseudotyped virus displayed in Fig.2 by one-way analysis of variance (ANOVA) test using GraphPad Prism 9.3.1. A *p-*value of less than 0.05 was considered significant. *, *p* < 0.05; **, *p* < 0.01; ***, *p* < 0.001; ****, *p* < 0.0001; ns (not significant), *p* ≥ 0.05.
